# Functional characterization of a novel plant growth-promoting rhizobacterium enhancing root growth and salt stress tolerance

**DOI:** 10.1038/s41598-025-14065-1

**Published:** 2025-08-19

**Authors:** Sanghee Lee, Young Kook Kim, Hualin Nie, Jongmin Ahn, Nayoung Kim, Seo-Rin Ko, Ah-Hyeon Choi, Hayoung Kwon, Yuxin Peng, Suk-Yoon Kwon, Ah-Young Shin

**Affiliations:** 1 Plant Systems Engineering Research Center, Korea Research Institute of Bioscience and Biotechnology (KRIBB), Daejeon, 34141 Republic of Korea; 2 Department of Functional Genomics, KRIBB School of Bioscience, University of Science and Technology (UST), 217 Gajeong-ro ,Yuseong-gu, Daejeon, 34113 Republic of Korea; 3 Natural Product Research Center, KRIBB, 30-Yeongudanji-ro, Ochang-eup, Cheongwongu, Cheongju-si, Chungbuk 28116 Republic of Korea; 4 Biological Resource Center, Korean Collection for Type Cultures (KCTC), KRIBB, Jeongeup, 56212 Republic of Korea; 5 Department of Biological Sciences, Sungkyunkwan University, 2066 Seobu-ro, Suwon, 16419 Republic of Korea

**Keywords:** Adventitious roots, Auxin, Lateral roots, Plant growth–promoting rhizobacteria (PGPR), Pseudomonas, Salt stress tolerance, Whole-genome sequencing (WGS), Microbiology, Physiology, Plant sciences, Environmental sciences

## Abstract

Plant growth–promoting rhizobacteria (PGPR) are soil microorganisms through which phytohormones and other bioactive compounds are produced, thereby enhancing plant growth and stress tolerance. In this study, a novel PGPR strain was identified from the rhizosphere of *Lycium chinense* seedlings, which produce protein-rich fruit. Whole-genome sequencing and annotation revealed that the genome of this strain, designated *Pseudomonas* sp. A-2, consists of a 6.65-Mb circular chromosome with 5,980 predicted protein-coding sequences. Comparative genomic analysis classified the strain within the genus *Pseudomonas*. The A-2 strain genome encodes proteins involved in indole-3-acetic acid (IAA) biosynthesis and signaling pathways, which was validated through IAA detection assays and quantitative analyses. Plant growth rates were significantly enhanced by the A-2 strain treatment, with increases of 3-fold in *Arabidopsis*, 1.5-fold in tobacco, and 1.35-fold in peanut. In *Arabidopsis thaliana*, expression of key genes associated with lateral and adventitious root formation was induced by the A-2 strain treatment, including *ARFs*, *AMI1*, *TAA1*, *YUCs*, *IBRs*, *TOB1*, and *ECH2*. Moreover, enhanced tolerance to salt stress was conferred by the A-2 strain treatment, as evidenced by improved biomass accumulation, chlorophyll content, antioxidant enzyme activity, and reduced lipid peroxidation. Levels of total soluble sugars, including trehalose, were elevated in the A-2 strain treated plants, suggesting a role in osmotic adjustment under stress. The plant growth–promoting and stress-alleviating properties of *Pseudomonas* sp. A-2 highlight its potential application as an effective biological agent for sustainable agriculture.

**Supplementary Information:**

The online version contains supplementary material available at 10.1038/s41598-025-14065-1.

## Introduction

Fertilizers have long been indispensable in modern agriculture for enhancing crop productivity and meeting global food demands. However, their excessive or imbalanced use has raised concerns over environmental sustainability, soil degradation, and reduced crop quality. As a promising eco-friendly alternative, plant growth–promoting rhizobacteria (PGPR) have been increasingly studied due to their ability to improve plant performance by interacting with root systems without establishing obligate symbiotic relationships^1,2^.

Various types of PGPR have been studied for their abilities to produce indole-3-acetic acid (IAA), synthesize siderophores, produce 1-aminocyclopropane-1-carboxylate deaminase (ACC) deaminase (EC 3.5.99.7), solubilize phosphate, fix nitrogen, and suppress fungal pathogens. Reported PGPR genera include *Azospirillum*^3^*Bacillus*^4^*Brevundimonas*^5^*Enterobacter*, *Klebsiella*,* Serratia*^6,7^*Pseudomonas*^8,9^*Rhizobium*^9,10^and *Streptomyces*^11^. In addition, PGPR are known to contribute to plant protection^12^improve soil structure^13^utilize soil nutrients to stimulate plant growth^14^produce plant growth regulators^15^and control or inhibit plant pathogens^16,17^. The plant growth–promoting effects of PGPR have been documented in numerous studies involving various plant species, including *Arabidopsis*^18,19^pea^20^rice^21,22^soybean^23^tobacco^24^tomato^25^and wheat^26^.

PGPR have also been shown to participate in the biosynthesis of auxins^27,28^. Auxins are key regulators of plant development, modulating root and shoot architecture, lateral root (LR) formation, and tropic responses^29–34^. Tryptophan is the principal precursor of IAA^35–38^. IAA is synthesized from tryptophan through four distinct pathways: (1) the indole-3-acetamide (IAM) pathway, (2) the indole-3-pyruvic acid (IPA) pathway, (3) the tryptamine (TAM) pathway, and (4) the indole-3-acetaldoxime (IAOx) pathway^39–44^. The involvement of various soil microorganisms in IAA synthesis has been demonstrated both in pure cultures and soil environments^45–47^. It has also been reported that some PGPR harbor the *iaaM* gene, enabling high-level IAA production under specific environmental conditions^48,49^. Auxin-producing PGPR (e.g., *Bacillus* and *Azotobacter*) have been shown to significantly enhance crop yields and growth traits such as root and shoot development in various species, including maize, cucumber, and *Arabidopsis*^19,50–52^.

In addition to promoting growth under optimal conditions, PGPR have been recognized for their capacity to alleviate abiotic stress in plants. Salt stress is a major agricultural constraint, leading to osmotic imbalance, ion toxicity, and oxidative damage^53^. Certain salt-tolerant PGPR (ST-PGPR) have been shown to mitigate the effects of salinity by enhancing antioxidant capacity, improving osmotic adjustment, and modulating stress-responsive gene expression^54,55^. Moreover, PGPR have been reported to confer tolerance to other abiotic stresses, such as osmotic and drought stresses, which similarly impair plant growth by inducing water deficit, oxidative stress, and metabolic imbalance^56,57^. Through mechanisms such as the accumulation of compatible solutes, activation of antioxidant systems, and modulation of stress-related signaling pathways, PGPR are known to contribute to enhanced plant resilience under these challenging environmental conditions^58^.

While many studies have focused on the phenotypic effects of PGPR, relatively few have addressed the genetic basis of their functional traits. Whole-genome sequencing (WGS) has enabled comprehensive characterization of microbial genomes and has revealed key genes associated with PGPR functions, including nitrogen metabolism, hormone production, and stress resistance^59–63^. The integration of WGS with physiological and molecular analyses is considered essential to understand how PGPR influence plant development at the systems level.

In this study, a novel PGPR strain was isolated from the rhizosphere of *Lycium chinense*, and its ability to promote plant growth and enhance salt stress tolerance was investigated. Auxin-related activity was assessed, and effects on physiological parameters and gene expression were evaluated in multiple plant species. Through a multidisciplinary approach combining genomics, transcriptomics, and plant physiology, the functional role of this strain was elucidated, and its potential as a multifunctional biofertilizer for sustainable agriculture was explored.

## Materials and methods

### Bacterial strain isolation and DNA Preparation

Soil samples were collected from the rhizosphere of *L. chinense* located in Dongan-ri, Okcheon-eup, Okcheon Country, Chungcheongbuk-do, Republic of Korea (36.31°N, 127.59°E). For each sample, 1 g soil, including roots, was mixed with 9 mL distilled water and serially diluted 3 times at a 1:10 ratio, resulting in 1:10, 1:100, and 1:1000 dilutions. These dilutions were then spread onto various media: Bennet agar (1 g/L yeast extract, 1 g/L peptone B, 2 g/L tryptone, 10 g/L dextrose, 30 g/L bacto agar, pH, 7.3 ± 0.1), potato dextrose agar (PDA; 24 g/L potato dextrose broth, 30 g/L bacto agar, pH 5.6 ± 0.1), glucose yeast peptone (2 g/L yeast extract, 5 g/L peptone, 0.5 g/L MgSO_4_·7H_2_O, 1 g/L KH_2_PO_4_, 20 g/L glucose, 10 g/L bacto agar, pH 5.6 ± 0.1), or yeast glucose (5 g/L yeast extract, 10 g/L glucose, 10 g/L bacto agar, pH 5.6 ± 0.1). Plates were incubated at 28, 30, or 37 °C for 2 days. Microorganisms were isolated based on the morphological characteristics of colonies and grown in pure cultures. A single colony of a novel strain was cultured in liquid PDA medium at 30 °C for 24 h with shaking at 180 rpm. The cultured strain was stored as a 25% glycerol stock at − 80 °C. To select promising microbial candidates, colonies that inhibited the growth of neighboring microbes were prioritized, as these were presumed to exhibit competitive or antagonistic traits. Several isolates were shortlisted based on this criterion and subsequently subjected to preliminary plant growth promotion assays. A single strain that consistently promoted plant growth was selected for further experiments.

Total genomic DNA was extracted using a Quant-iT™ PicoGreen^®^ dsDNA Kit (Cat. No. P7589, Thermo Fisher Scientific, Waltham, MA, USA), following the manufacturer’s instructions. Genomic DNA integrity was assessed via 0.7% agarose gel electrophoresis, and DNA purity was evaluated using a NanoDrop UV–Vis Spectrophotometer (Cat. No. ND-2000, Thermo Fisher Scientific). DNA concentrations were quantified using a Qubit dsDNA HS Quantification Assay Kit (Cat. No. Q32854, Thermo Fisher Scientific) and a Qubit 4 Fluorometer (Cat. No. Q33238, Thermo Fisher Scientific). For sequencing, 4 µg DNA was used, and sequencing was conducted using both Illumina (San Diego, CA, USA) and Oxford Nanopore Technologies (ONT; Oxfordshire, UK) platforms.

### Bacterial strain sequencing and assembly

Libraries for long-read sequencing were prepared with end repair, dA-tailing, barcode ligation, and adapter ligation using the NEBNext^®^ Ultra™ End Repair/dA-Tailing Module [Cat. No. E7546, New England Biolabs Co. (NEB), Ipswich, MA, USA], FFPE Repair Mix NEBNext Quick Ligation Module (Cat. No. E6056, NEB), and Native Barcoding Kit (Cat. No. SQK-NBD114.24, ONT). Ligated DNA was purified, and long-read sequencing was conducted using a MinION Mk1C device R10.4.1 (Cat. No. MIN-101 C, ONT) with a SpotON Flow Cell (Cat. No. FLO-MIN114, ONT), according to the manufacturer’s instructions and managed via MinKNOW software v4.2.5 (ONT). Adapter sequences were removed using Guppy basecalling and Porechop v0.2.4^64^ (https://github.com/rrwick/Porechop*).* For long contig assembly, the Flye v2.9.2-b1786^65^ program was used, and taxonomic classification was performed with Kraken2 v2.1.2^66^ (https://github.com/DerrickWood/kraken2*).* Homopolish v0.3.4^67^ was employed to correct sequencing errors. Genome assembly completeness was evaluated using Benchmarking Universal Single-Copy Ortholog (BUSCO) v5.4.4^68^. Circlator v1.5.5^69^ was used to position the dnaA gene at the start of the contig. Gene prediction and annotation were completed with PROKKA v1.14.6^70^.

Short-read sequencing was performed to refine bacterial strain genomic sequences. Bacterial strain genomic DNA paired-end libraries with 350-bp inserts were generated using the TruSeq Nano DNA High Throughput Preparation Kit (Cat. No. 20015965, Illumina) and sequenced at Macrogens Co. (Seoul, Korea) using Illumina Sequencing by Synthesis technology. SPAdes v3.15.0^71^ (http://cab.spbu.ru/software/spades/*)* was used for genome assembly.

### Comparative genomic analysis

Average Nucleotide Identity (ANI) and Genome-to-Genome Distance Calculator (GGDC) values were calculated to evaluate the genomic relatedness between the A-2 strain and closely related strains. ANI and GGDC analyses were performed using the standalone version of the Orthologous Average Nucleotide Identity tool (OAT) downloaded from^72^ (https://www.ezbiocloud.net/tools/orthoani). In addition, Average Amino Identity (AAI) was calculated using ezAAI v1.2.3 (https://github.com/endixk/ezaai), and digital DNA-DNA hybridization (dDDH) values were estimated using Genome-to-Genome Distance Calculator v3.0 (https://ggdc.dsmz.de/distcalc2.php). Comprehensive measures of overall genomic similarity were provided by these analyses to support species delineation of the A-2 strain.

### Scanning electron microscopy (SEM)

Samples were fixed in 2.5% paraformaldehyde–glutaraldehyde (4 °C, phosphate buffer, pH 7.2) for 4 h, followed by three washes with 0.1 M phosphate buffer (pH 7.2) for 10 min each. Subsequently, samples were post-fixed in 1% OsO_4_ (25 °C, 0.1 M phosphate buffer, pH 7.2) for 1 h, followed by several washes with 0.1 M phosphate buffer (pH 7.2) and dehydration in increasing ethanol concentrations. Substitution with isoamyl acetate and critical point drying were performed, and a 20-nm-thick coating was applied using an SC502 sputter coater. The specimens were observed at 5 kV using the FEI Quanta 250 FEG scanning electron microscope (FEI, Hillsboro, Oregon, USA).

### Measurement of IAA production by bacterial strains

Bacterial strains were grown at 30 °C for 1 day with shaking at 180 rpm. Cultures were grown in liquid PDA with or without 100 mg/L L-tryptophan (L-Trp). A 5 mL sample of cultured bacteria was centrifuged at 2,700 × *g* for 15 min at 4 °C. The supernatant (1 ml) was mixed with 2–3 drops of ortho-phosphoric acid and 4 mL of Salkowski’s reagent (1 mL 0.5 M FeCl_3_ dissolved in 50 mL 35% HClO_4_)^73^. Salkowski’s reagent allows for the detection of substances belonging to the indole class. Samples were incubated at 30 °C for 30 min in the dark, and absorbance was measured at 535 nm. The IAA concentration in each bacterial strain was determined using a calibration curve generated using synthetic IAA.

### Quantitative analysis of IAA production

Ultra-performance liquid chromatography (UPLC) analysis was conducted to confirm the identity of IAA produced by bacterial strain. Cells were isolated from a 5 mL bacterial culture by centrifugation for 15 min at 2,700 × *g* at 4 °C. The pH of the supernatant was adjusted to 2.8 with 1 M HCl, followed by three successive extractions with ethyl acetate. The dried extract was dissolved in 1 mL methanol. Samples were analysed using ACQUITY™ UPLC I-Class (Waters, Milford, MA, USA) and an ACQUITY UPLC^®^ BEH C_18_ column 1.7 μm (2.1 mm × 100 mm, Waters). IAA was separated using a mobile phase of acetonitrile with 0.1% formic acid (v/v) and water. The gradient was initiated at 5% acetonitrile at a flow rate of 0.4 mL/minute, increasing to 40% over the first 8 min and then to 100% over 0.3 min. The injection volume for all samples was 1 µL, and the column temperature was maintained at 35 °C. The UPLC system was coupled with a Waters XEVO-QTOF-mass spectrometer (Micromass, Manchester, UK) equipped with an electrospray ionization scan mode. The mass conditions for multiple reaction monitoring mode were as follows: capillary voltage, 2.3 kV; source temperature, 110 °C; desolvation temperature, 350 °C; desolvation gas flow, 800 L/hour; and cone voltage, 40 V.

### Bacterial culture and plant growth experiments

Bacterial strains were cultured on PDA medium at 30 °C for 24 h. The optical density at 600 nm (OD_600_) was measured, and the culture was adjusted to an OD_600_ of 0.5, equivalent to 3–4 × 10^6^ colony-forming units mL^− 1^. In vitro experiments were conducted on *Arabidopsis thaliana* (ecotype Columbia-0). Seeds were sterilized by treatment with 70% ethanol for 1 min, followed by a solution of 1% sodium hypochlorite mixed with 0.05% Triton X-100 for 10 min, and rinsing eight times with sterile triple-distilled water (3DW). After sterilization, seeds were cold-treated at 4 °C for 2 days, then sown on square dishes (120 × 120 mm) containing half-strength Murashige and Skoog (MS) medium supplemented with 20 g/L sucrose and 10 g/L phyto agar. A total of 20 seeds were sown on each plate, and plates were incubated in a growth chamber at 25 ± 0.2 °C with a 16-hour light/8-hour dark photoperiod. The germination rate was assessed 4 days after sowing, and 12 uniformly growing seedlings were selected for subsequent experiments. Bacterial strains were cultured for 24 h. To ensure even exposure of the root zone, 60 µL of bacterial suspension was divided into small droplets and spot-applied along the agar surface, with one droplet placed per grid cell beneath each *Arabidopsis* seedling. PDA liquid medium was used as the control. The fresh weights of leaves and roots were measured at 1, 3, 5, and 7 days after treatment (DAT). Samples were collected in clear, 1.5 mL tubes and stored at − 80 °C until further analysis. In hypocotyl excision experiments, 5-day-old seedlings grown on germination medium were excised at the hypocotyl–root junction to induce adventitious root (AR) formation and bacterial strains were applied as described above. The fresh weights and data on AR formation were collected at 10 DAT.

*Nicotiana tabacum* cv. Xanti was also assessed. Seeds were sterilized and sown on square plates containing medium, as described for *A. thaliana*. A total of 12 seeds were sown per plate, and plates were incubated under the same growth conditions as described for *A. thaliana*. The germination rate was assessed after 10 days, and 10 uniformly germinated seedlings were selected for experiments. For *N. tabacum*, 100 µL of bacterial suspension was applied to the center of the agar surface and allowed to absorb before placing the seedlings. Due to the larger seedling size and longer treatment period, full-surface application led to occasional overgrowth and tissue damage. Central application was therefore used to minimize contamination while maintaining effective exposure. Data on fresh weights and root development were collected at 14 DAT.

Peanut (*Arachis hypogaea*) seeds were soaked in water for 1 day, placed on wet tissue for 2 additional days, and subsequently planted in 32-plug trays. The seedlings were grown for 4 days additional days (7 days total after sowing) before bacterial treatments began. A microbial solution (bacterial culture diluted 1:5 with 3DW) was applied by adding 25 mL microbial solution to each tray compartment at 3-day intervals over 14 total days. The control group was treated with PDA medium diluted 1:5 with 3DW.

### Salinity stress treatment and physiological measurements

#### Plant growth condition

For the salinity stress treatment, 4-day-old *A. thaliana* seedling were transferred to germination medium supplemented with varying concentrations of NaCl (0, 100, and 150 mM). The plates were incubated for 14 days under the same growth conditions as in the previous experiment, following bacterial treatment. In the A-2 strain treated group, bacterial suspension (OD_600_ = 0.1) was evenly applied to the surface of the medium and allowed to absorb. Each treatment group contained 12 seedlings per plate, with three seedlings considered as one biological replicate.

#### Chlorophyll quantification

Chlorophyll content was measured to assess the physiological status of leaf tissue under stress. Approximately 10–40 mg of fresh leaf tissue was weighed and incubated in 1 mL of absolute ethanol for 24 h in the dark at room temperature. Following extraction, the supernatant was separated by centrifugation, and absorbance was measured at 645 nm and 663 nm using a UV–Vis spectrophotometer. Total chlorophyll content was calculated using the following equation : Chlorophyll (µg mg⁻¹ FW) = (20.2 × A₆₄₅ + 8.02 × A₆₆₃) / fresh weight (mg). Each treatment included three biological replicates, and each replicate consisted of three seedlings.

#### Superoxide dismutase (SOD) activity

Fresh aerial tissues were immediately frozen in liquid nitrogen, finely ground with a mortar and pestle, and homogenized in cold lysis buffer (50 mM potassium phosphate, 0.1 mM EDTA, 0.5% Triton X-100, pH 7.4). The homogenate was centrifuged at 12,000 × g for 5 min at 4 °C, and the resulting supernatant was used for protein quantification and antioxidant enzyme assays. SOD activity was measured using a commercial SOD Assay Kit (Sigma-Aldrich, Cat. No. MAK528, St. Louis, MO, USA) according to the manufacturer’s instructions. The assay was performed in a 96-well microplate format. Briefly, 20 µl of the crude enzyme extract was added to each well, followed by 160 µl of Working Solution and 20 µl of Xanthine Oxidase Solution. After adding all reagents, the absorbance at 440 nm was immediately measured using a microplate reader (Tecan Group Ltd., Männedorf, Switzerland), without additional incubation, following the kit protocol. One unit of SOD activity was defined as the amount of enzyme causing a 50% inhibition of the reduction of WST-1 reagent, as described in the kit protocol. All experiments were conducted with three biological replicates, each containing three seedlings.

#### Free proline content determination

Proline content was quantified according to the acid-ninhydrin method. Fresh shoots (~ 40 mg) were homogenized in 3% sulfosalicylic acid and centrifuged at 12,000 × g for 6 min. The resulting supernatant was reacted with acid ninhydrin reagent (1.25% ninhydrin in glacial acetic acid and 6 M phosphoric acid) and incubated at 100 °C for 1 h. After cooling, the chromophore was extracted with toluene, and absorbance was read at 520 nm. Proline concentrations were calculated from a standard curve using L-proline and expressed as µmol g⁻¹ fresh weight (FW).

#### Lipid peroxidation assay

Malondialdehyde (MDA) levels were measured as an indicator of lipid peroxidation using the Lipid Peroxidation (MDA) Assay Kit (Abcam, ab118970, Cambridge, UK), following the manufacturer’s protocol. Briefly, ground shoot tissue was homogenized in lysis solution centrifuged, and the supernatant was used for the assay. Absorbance was measured at 532 nm. MDA levels were calculated based on a standard curve generated using the provided MDA standard.

#### Quantification of total soluble sugar (TSS) and Trehalose

Total soluble sugars were quantified using the phenol–sulfuric acid method described by Dubois et al.^74^. Briefly, 200 µL of the sample extract was mixed with 200 µL of 5% phenol solution and 1 mL of concentrated sulfuric acid. The mixture was incubated at room temperature for 30 min, and absorbance was measured at 490 nm using a UV–Vis spectrophotometer.

Trehalose content was determined using the anthrone–sulfuric acid method as described by Magazin and Popovic^75^with trehalose as a standard. Absorbance was measured at 620 nm after color development, and results were expressed as mg trehalose per gram fresh weight (mg/g FW).

### RNA isolation and quantitative reverse transcription polymerase gain reaction (RT-qPCR) analysis

Total RNA was extracted from *A. thaliana* root samples collected from three independent experiments: at 1, 3, 5, and 7 DAT; at 10 DAT; and at 14 DAT under salt stress conditions (0, 100, and 150 Mm NaCl), using the HiYield™ Total RNA Mini Kit (Cat. No. YRP100, RBC, Banqiao, Taiwan). RNA concentration and purity were assessed with a NanoDrop 2000 spectrophotometer (Thermo Fisher Scientific, Waltham, MA, USA). cDNA was synthesized by reverse transcription of 1 µg total RNA with the EcoDry™ Premix Oligo dT Kit (Cat. No. 639543, Takara Bio Inc., Kyoto, Japan). In a total reaction volume of 25 µL, 1 µL cDNA template was combined with 2×TB Green Fast qPCR Mix (Takara Bio Inc.), and auxin-related gene expression levels were quantified by RT-qPCR, according to the manufacturer’s instructions. PCR samples were amplified using a Thermal Cycler Dice Real-Time PCR system (Takara Bio Inc.). The RT-qPCR cycling conditions were as follows: initial denaturation at 95 °C for 30 s, followed by 40 cycles of 95 °C for 5 s and 60 °C for 10 s. A melting curve analysis was performed with dissociation steps at 95 °C for 15 s, 60 °C for 30 s, and 95 °C for 15 s. The PCR efficiency for each primer pair was calculated based on the equation E = [10^(−1/slope)^ − 1] × 100%, using the slope of the standard curve (maximum CT value of < 40). The value obtained from this equation should be in the range of 90–110%^76^. Relative gene expression levels were calculated using the comparative threshold cycle (2^−ΔΔCt^) method and normalized to the expression of *actin2* mRNA as an internal reference. Primers (Supplementary Table 6) were designed using GenScript Real-time PCR (TaqMan) Primer and Probes Design Tool (https://www.genscript.com/tools/real-time-pcr-taqman-primer-design-tool*)*, with lengths of 20–26 nucleotides, a target product size of 50–400 bp, and annealing temperatures ranging from 52 °C to 60 °C. RT-qPCR experiments were conducted with three biological replicates.

###  Statistical analysis

For all experiments, data were analysed by Student’s t-test with equality of variance, and differences between samples treated under control conditions or with bacterial strains were considered significant at *P* values equal to or less than 0.05. All experiments were performed with at least three biological replicates.

## Results and discussion

### Microorganism Preparation and whole-genome sequencing

**Fig. 1:**
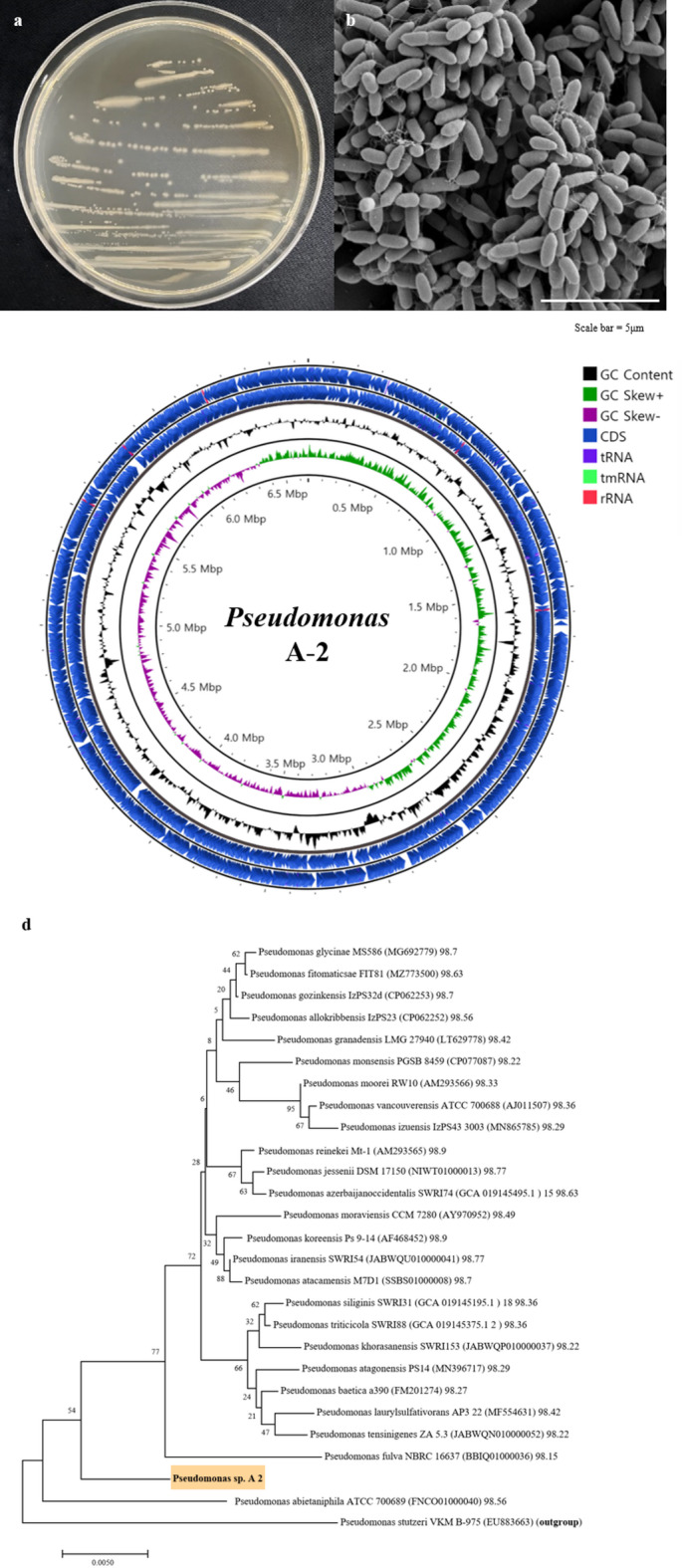
Characteristics of *Pseudomonas* sp. A-2. (**a**) Circos plot showing the completed assembly and annotation data generated using Proksee (CGview). From the outer to the inner circle : coding domain sequence (CDS), tRNA genes, tmRNA genes, rRNA genes, GC content, GC skew, base pairs (bp). (**b**) Morphology of the A-2 strain colonies on an agar plant after 24 h of growth at 30 °C. (**c**) Scanning electron microscopy image of the A-2 strain grown for 24 h at 30 °C, shown at 10,000x magnification. (**d**) Phylogenetic tree of *Pseudomonas* species, based on whole-genome sequencing of the 16S rRNA region, showing the relationships among various strains within the genus. The species highlighted with a pale orange and labeled as ‘*Pseudomonas* sp. A-2’ indicates the specific strain of interest. The tree was generated in CLC Main Workbench 22 using the neighbor-joining method with 1,000 bootstrap replicates. The scale bar represents 0.005 substitutions per nucleotide position.

Microorganisms were isolated from the rhizosphere surrounding *L. chinense* seedlings based on colony morphology. One bacterial strain was designated as ‘A-2’, which formed round, mucilaginous, pale-yellow colonies on PDA agar plates (Fig. 1a). SEM analysis revealed cylindrical cells with flagella, indicating motility (Fig. 1b).

Whole-genome sequencing (WGS) was performed using Nanopore (1.26 Gb, 188.6× coverage) and Illumina (2.48 Gb, 376.4× coverage) platforms. The assembled genome was found to comprise a single circular chromosome of 6.6 Mb (Fig. 1c), containing 5,896 protein-coding genes, 16 rRNA genes, 67 tRNA genes, and 1 tmRNA gene. BUSCO analysis confirmed 98.7% completeness (772 genes) (Table 1). Genome annotation revealed the presence of the tryptophan-2-monooxygenase (*iaaM*)^77^essential for IAM-mediated IAA biosynthesis, along with other IAA-related genes, including *trpA–F* (encoding tryptophan biosynthesis enzymes) and enoyl-CoA hydratase 1 *(ech1)* (Table 2). The EggNOG-based functional categorization is shown in Supplementary Fig. 4.

A phylogenetic tree based on the 16S rRNA gene sequence placed the A-2 strain within the genus *Pseudomonas* (Fig. 1d). Comparative genomic analyses with ten phylogenetically related *Pseudomonas* strains revealed ANI values ranging from 75.0 to 85.03%, below the species threshold of 95%^78^ (Supplementary Fig. S3a). Consistently, dDDH values calculated via GGDC ranged from 0.14 to 0.20 (Supplementary Fig. S3b), well below the 70% species delineation cutoff. Hierarchical clustering of ANI and dDDH values (Supplementary Fig. S3c and d) further supported the classification of the A-2 strain as a novel genome species within *Pseudomonas*.

### Characterization of IAA production by Pseudomonas sp. A-2

**Fig. 2:**
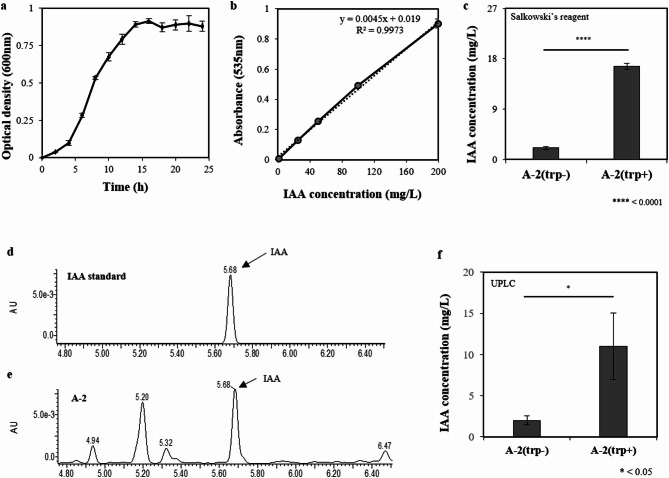
Analysis of indole-3-acetic acid (IAA) extraction from *Pseudomonas* sp. A-2. (**a**) Growth curve of the A-2 strain grown in liquid potato dextrose agar (PDA) at 30 °C and 200 rpm over 24 h, with optical density values measured every 2 h. (**b**) Standard curve of synthetic IAA concentrations with Salkowski’s reagent. (**c**) IAA concentration of the A-2 strain isolates grown in the presence and absence of tryptophan, measured by spectrophotometry. Blank: liquid PDA medium mixed with Salkowski’s reagent. (**d**) Chromatogram of synthetic IAA (12.5 ppm). (**e**) Chromatogram of IAA extracted from the A-2 strain grown in the presence of 100 mg/mL tryptophan, showing a retention time at 280 nm. (**f**) Ultra-performance liquid chromatography was used to measure IAA concentrations following the A-2 strain isolate growth in the presence and absence of tryptophan. *P < 0.05; ****P < 0.0001. The results are presented as the mean ± standard deviation (*n* = 3).

As *Pseudomonas* species are known to produce IAA, the newly isolated the A-2 strain was evaluated for its IAA production potential following the identification of IAM-related genes through genome annotation. Growth curve analysis revealed that maximum IAA production was achieved during the stationary phase, which was reached after approximately 20 h of culture (Fig. 2a). In the presence of 100 mg/L L-Trp, the strain produced 16.67 mg/L IAA, as measured by Salkowski’s reagent-based colorimetric assay (Fig. 2b, c, and Supplementary Table 2). The retention time for bacterially derived IAA at 280 nm was determined to be 5.68 min, which corresponded to that of the synthetic IAA standard (Fig. 2d and e).

For more accurate quantification, UPLC analysis was performed using individual standard curves, and the IAA concentration was determined to be 10.98 mg/L (Fig. 2f). Although the values obtained by Salkowski’s method and UPLC did not completely coincide, the difference between the two measurements was not substantial, supporting the validity of the overall IAA production estimation. Given that the Salkowski’s assay is a colorimetric screening tool with limited specificity, the UPLC-derived value is considered to be more accurate and reliable. Notably, although some microbes require L-Trp as a precursor for auxin biosynthesis^79 ^the A-2 strain was capable of producing IAA even in the absence of L-Trp (Fig. 2c, f, and Supplementary Fig. 6), indicating the presence of an L-Trp independent biosynthetic pathway.

### Effect of pseudomonas sp. A-2 on plant growth and lateral root formation

**Fig. 3:**
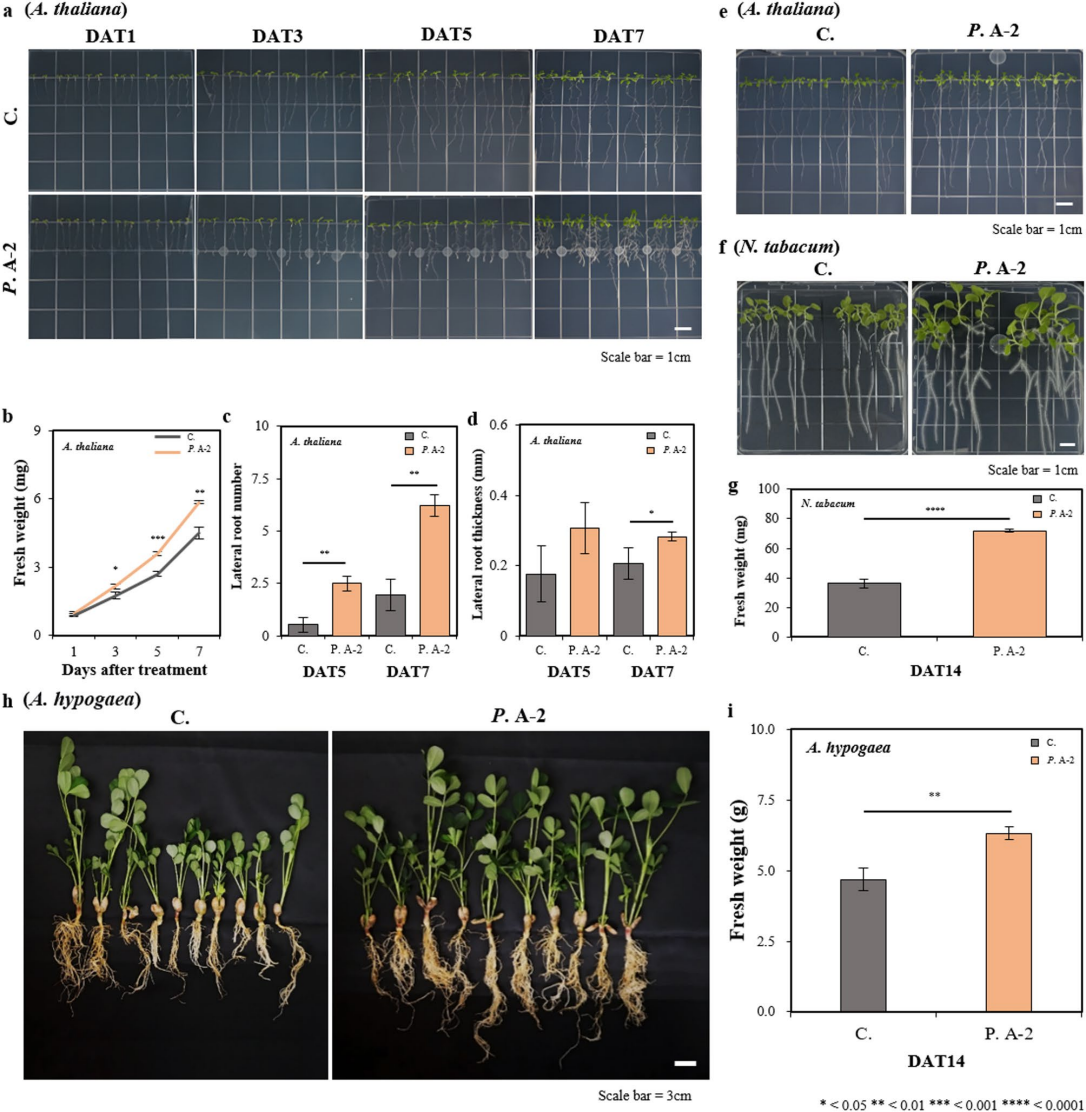
Plant growth response to treatment with *Pseudomonas* sp. A-2. (**a**) Plant morphology of *A. thaliana* was observed 1, 3, 5, and 7 days after treatment (DAT) with a suspension of the A-2 strain applied at the middle of the vertical plate. (**b**) Fresh weight of *A. thaliana* was measured DAT 1, 3, 5, and 7 with the A-2 strain. (**c**) Lateral root number and (**d**) root thickness were measured DAT5 and DAT7 with the A-2 strain. (**e**) Growth differentiation based on the treatment position of the A-2 strain: growth was assessed DAT7 with a suspension solution applied at the top of the vertical plate. (**f**) Morphology of *N. tabacum* was observed DAT14 with a suspension of the A-2 strain applied at the middle of the vertical plate. (**g**) Fresh weight of *N. tabacum* was measured DAT14 with the A-2 strain. (**h**) Plant morphology of peanut plants DAT14 with a suspension of the A-2 strain applied to the soil. (i) Fresh weights of peanut plants were measured DAT14. Significant differences between control (C.) and *Pseudomonas* sp. A-2 treated plants (*P*. A-2) were evaluated using Student’s t-test : *P < 0.05; ** *P* < 0.01; *** *P* < 0.001; **** *P* < 0.0001. The results are presented as the mean ± standard deviation (*n* = 3).

The effects of the A-2 strain treatment on *A. thaliana* were assessed by measuring fresh weight, LR number, and LR thickness between DAT1 and DAT7 (Fig. 3). Compared to untreated plants, the A-2 strain treatment led to a 1.3-fold increase in fresh weight, a 3.2-fold increase in LR number, and a 1.4-fold increase in LR thickness at DAT7 (Fig. 3a–d and Supplementary Table 3). LRs were first observed on day 3, and data collection was initiated on day 5. The A-2 strain, known to produce IAA, enhanced LR development near the treatment site while reducing primary root (PR) length, consistent with previous findings that auxin levels above 10⁻⁸ M suppress PR growth and stimulate LR formation^80^. However, when the A-2 strain was applied to the upper portion of the plant, PR length remained unaffected and LR formation resembled that of untreated controls (Fig. 3e). This suggests that the active compound is not a volatile organic compound (VOC), as VOCs are known to influence plant hormone levels^81^and high IAA levels can elevate ethylene, inhibiting root growth^82^. The absence of inhibitory effects implies that the IAA concentration produced by the A-2 strain is below the critical threshold.

Growth-promoting effects were also observed in *N. tabacum*, a model crop species frequently used in plant-microbe interaction studies. Application of the A-2 strain at the middle of the growth plate resulted in a 2-fold increase in total growth by DAT14, including elongation of both LR and PR (Fig. 3f, g, and Supplementary Table 4), whereas only PR elongation was noted in the control group.

Pot-based experiments were conducted to evaluated the effects of the A-2 strain on peanuts (*A. hypogaea*), a leguminous crop in which LR development and nodule formation originate from distinct cell types but are functionally interconnected, contributing to growth and pod development. Peanut sprouts also contain resveratrol, an antioxidant known for scavenging reactive oxygen species (ROS)^83,84^. Resveratrol is associated with hormonal pathways, including the biosynthesis and regulation of IAA and other auxins, while also scavenging ROS, providing a rationale for the experiment. Given that the A-2 strain enhances both root and shoot development, peanut was selected as a biologically and agronomically relevant system to evaluate whether root-promoting PGPR activity translates into increased biomass in a geocarpic legume where subterranean development is crucial for productivity. Compared with the untreated controls, the A-2 strain treated group exhibited enhanced shoot and root development and a 1.35-fold increase in total biomass (Fig. 3h and i).

### Quantitative measurement of gene expression

**Fig. 4:**
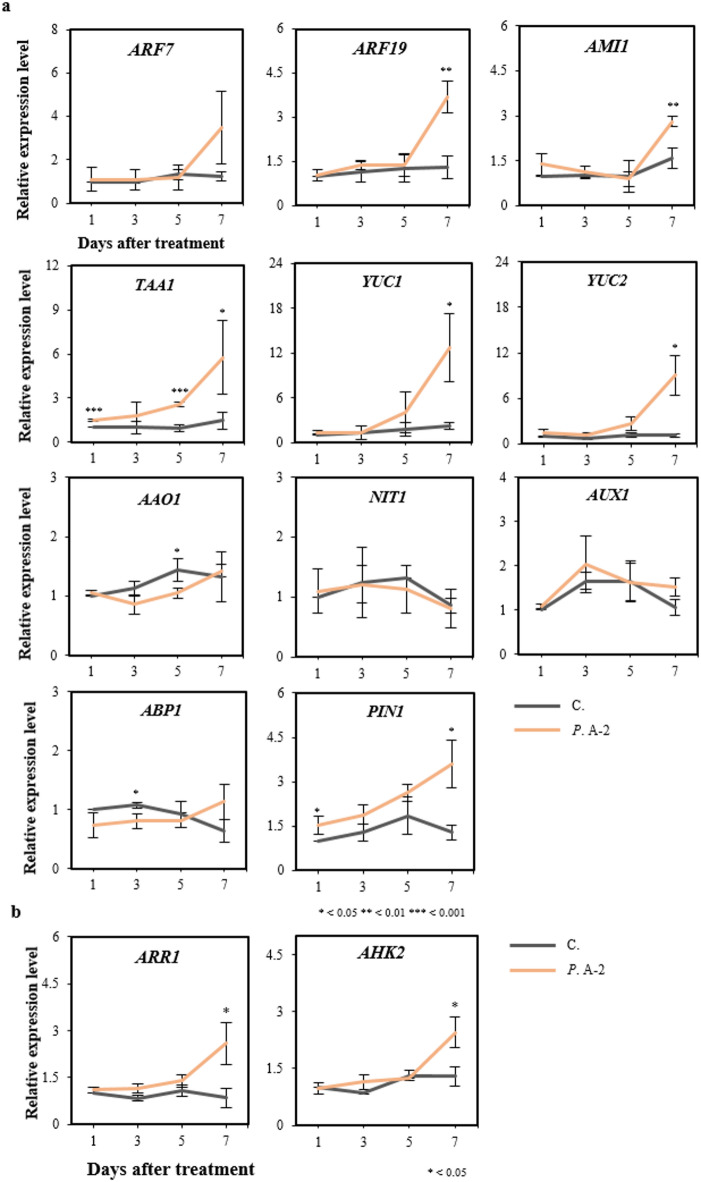
Expression analysis of auxin and cytokinin biosynthesis genes in *A. thaliana* using RT-qPCR. (**a**) Expression levels of genes in the tryptophan to IAA synthesis pathway during early plant growth. *A. thaliana* seedlings were cultured on germination medium, and roots were sampled 1, 3, 5, and 7 days after treatment (DAT) with a suspension of the A-2 strain applied at the middle of the vertical plate. (**b**) Expression levels of genes in the cytokinin biosynthesis pathway during early growth stages. *A. thaliana* seedlings were cultured on germination medium, and roots were sampled DAT1, 3, 5, and 7. Relative gene expression levels were normalized to the untreated control (without the A-2 strain) and calibrated for each respective day. Significant differences between control (C.) and *Pseudomonas* sp. A-2 treated plants (*P*. A-2) were evaluated using Student’s t-test : *P < 0.05, **P < 0.01, ***P < 0.001. The results are presented as the mean ± standard deviation (*n* = 3).

To evaluate whether auxin biosynthesis in *A. thaliana* roots was influenced by treatment with the A-2 strain, RT-qPCR was performed on root samples collected at various time points after treatment. The expression level of auxin response factor 19 (*ARF19*), a central regulator of the auxin signaling pathway, was not significantly altered until DAT5, but was markedly elevated by DAT7 in the A-2 strain treated plants (Fig. 4a). *ARF7* and *ARF19* are critical for^85,86^ and play precise roles in the initiation of LR formation^85,87^. Therefore, the observed increases in expression suggests that the A-2 strain modulated auxin signaling during later developmental stages, leading to enhanced transcription of relevent genes.

To elucidated the biosynthetic pathways contributing to IAA production, the expression of several IAA biosynthesis genes was assessed. These included *AMI1* (encoding amidase 1; EC 3.5.1.4) involved in the IAM pathway, and *TAA1* (encoding tryptophan aminotransferase of *Arabidopsis* 1; EC 2.6.1.27), *YUCCA1* (YUC, encoding Flavin monooxygenase; EC 1.14.13.168), and *YUC2* from the IPA pathway. The expression of these genes, representing both IAM and IPA pathways, showed 3–6-fold increases in the A-2 strain treated plants at DAT7 (Fig. 4a). *AMI1* is essential for auxin homeostasis and supports growth and stress tolerance^88^while *TAA1*, *YUC1*, and *YUC2* contribute to IAA synthesis via the IPA pathway, acting cooperatively^36^. Given that *TAA1* and *YUCCAs* are associated with the formation of auxin gradients essential for LR primordia (LRP) development, it is likely that the A-2 strain supplies an exogenous source of auxin during early LRP formation and stimulatneously promotes endogenous auxin biosynthesis.

Conversely, no significant changes were observed in *AAO1* (encoding *Arabidopsis* aldehyde oxidase 1; EC 1.2.3.1), a key enzyme in the TAM pathway, or in *NIT1* (encoding nitrilase 1; EC 3.5.5.1), involved in the IAOx pathway^89^suggesting that these pathways do not contribute to the incresed IAA levels observed (Fig. 4a).

IAA perception is mediated by auxin-binding protein (*ABP*) receptors, while its cellular import and export are regulated by auxin influx carriers (*AUX*) and efflux carriers (*PIN*), respectively. In this study, the expression of *AUX1*, which facilitates auxin import from vascular tissues and supports LR formation^90,91^remained unchanged following the A-2 strain treatment, indicating that auxin uptake mechanisms were not affected (Fig. 4a). Similarly, *ABP1*, which functions in auxin signaling rather than polar transport, showed no significant expression change. In contrast, *PIN1*, encoding a key auxin efflux carrier required for root development^92,93^was progressively upregulated, suggesting that the A-2 strain treatment may influence auxin distribution within root tissues. Although gain-of-function mutants of *ABP1* have been shown to disrupt *PIN* polarity^94^the mechanism underlying the observed increase in *PIN1* expression remains unclear and may involve complex auxin regulatory networks.

To further examine the impact of the A-2 strain treatment on cytokinin pathways in *A. thaliana* roots, RT-qPCR was performed on root samples collected at multiple time points (Fig. 4b). Among the cytokinin signaling genes analysed, only *Arabidopsis* response regulator 1 (*ARR1*) was significantly upregulated (2.5-fold at DAT7). As cytokinins regulate root architecture, and *ARR1* mediates cytokinin signaling to repress auxin activity in quiescent center cells, its upregulation suggests enhanced cytokinin-meditaed signaling that may promote cell division and influence differentiation^95^. Additionally, *AHK2*, encoding a cytokinin receptor known to suppress LR formation in wild-type plants, was also upregulated at DAT7. However, no significant changes in LR induction were observed, implying that the increse in *AHK2* expression likely reflects elevated cytokinin levels or sensitivity induced by the A-2 strain treatment, rather than a direct effect on LR development.

### Adventitious root formation effects induced by pseudomonas sp. A-2 treatment

**Fig. 5:**
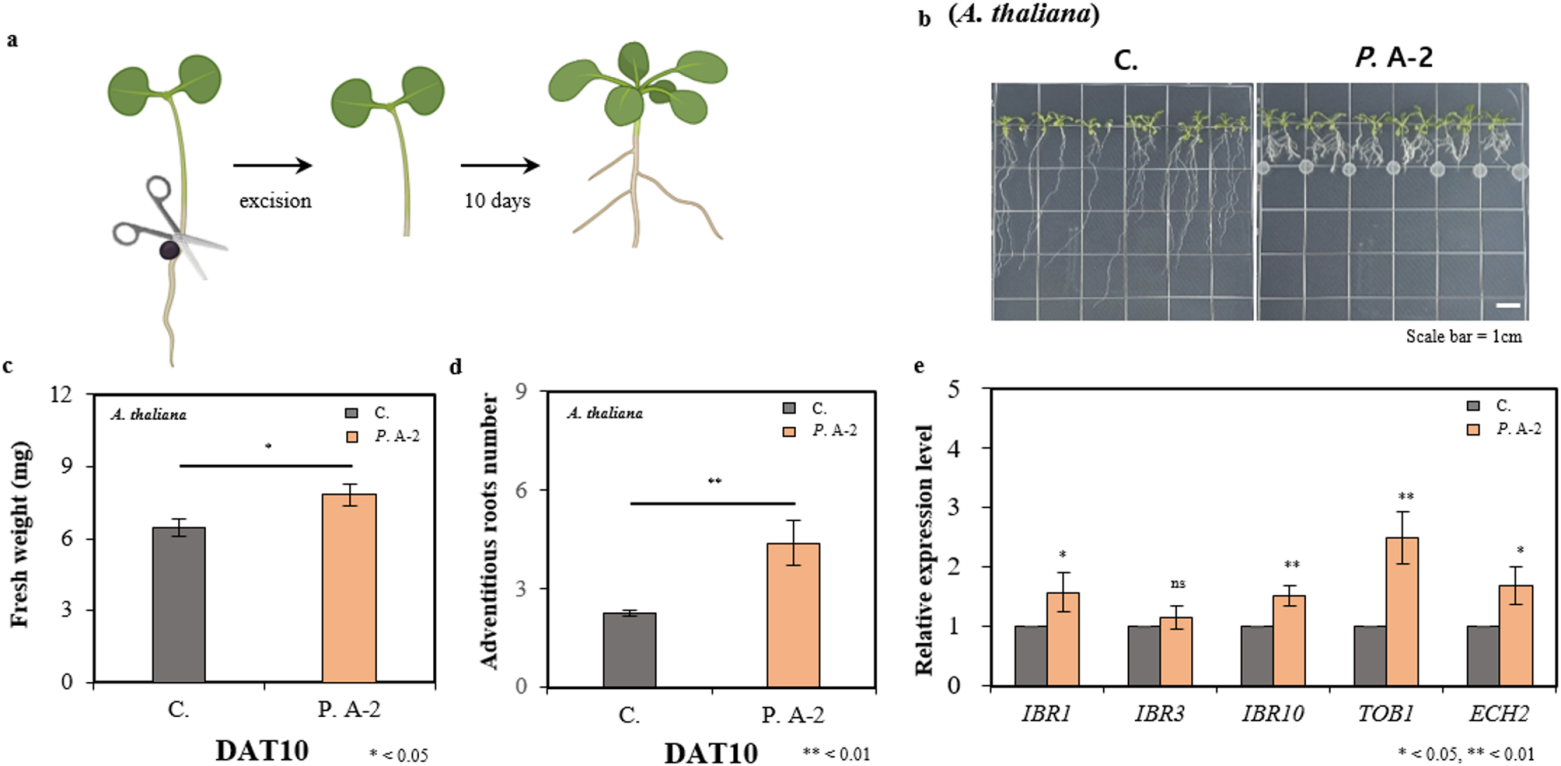
Regeneration of adventitious roots in *A. thaliana* induced by treatment with *Pseudomonas* sp. A-2. (**a**) In the assay for adventitious root formation induced by excision, the roots of 5-day-old seedlings were excised above the root–shoot junction and the medium was treated with the A-2 strain suspension. Observations and data collection were performed 10 days after hypocotyl excision and treatment (DAT) with the A-2 strain suspension on a vertical plate with *A. thaliana*. (**b**) Plant morphology, (**c**) fresh weight, and (**d**) number of adventitious roots were assessed DAT10. (**e**) Expression levels of genes involved in the IBA-to-IAA conversion pathway in *A. thaliana* were assessed using RT-qPCR in roots sampled DAT10. Relative gene expression levels were normalized to the untreated control (without the A-2 strain) and calibrated. Significant differences between control (C.) and *Pseudomonas* sp. A-2 treated plants (*P*. A-2) were evaluated using Student’s t-test : *P < 0.05, **P < 0.01. The results are presented as the mean ± standard deviation (*n* = 3).

IAA production by the A-2 strain was confirmed, and its treatment was shown to enhance LR formation and overall growth in *A. thaliana*. Auxins, particularly IAA, are recognized as essential for both LR and AR formation. In plants, indole-3-butyric acid (IBA), a precursor of IAA, is converted into IAA, which is known to regulate AR development by triggering cellular reorganization in the hypocotyl to establish AR meristems^96^. To evaluate the effect of the A-2 strain treatment on AR formation, hypocotyl–root junctions of 5-day-old seedlings were excised, and the basal regions were treated with the A-2 strain (Fig. 5a). As a result, a 1.2-fold increase in fresh weight and a 2-fold increase in AR number were observed compared to untreated controls (Fig. 5b-d).

To determine whether the conversion of IBA to IAA was affected by the A-2 strain treatment, RT-qPCR analysis was performed. The IBA transporter *TOB1*, which is responsible for the vacuolar sequestration of IBA and restriction of AR formation^97^was found to be upregulated 2.5-fold in the A-2 strain treated plants. This expression pattern was consistent with the upregulation of cytokinin-responsive genes such as *ARR1* and *AHK2*, both of which have been reported to induce *TOB1* expression. Nevertheless, AR formation was significantly enhanced by the A-2 strain treatment, indicating that *TOB1*-mediated repression of AR formation was not sufficient to counteract the promotive effects of the treatment.

Genes encoding key enzymes involved in IBA-to-IAA conversion, including *ECH2* (encoding enoyl-CoA hydratase 2; EC 4.2.1.17) and the IBA response (*IBR*) proteins (*IBR1*, *IBR3*, and *IBR10*)^98–101^were also examined. The expression of *IBR1*, *IBR10*, and *ECH2* was found to be upregulated, whereas no significant change was observed in *IBR3* expression. These results suggest that AR formation was promoted by the A-2 strain treatment through the enhancement of IBA-to-IAA conversion and increased auxin biosynthesis, despite concurrent activation of cytokinin signaling pathways.

**Table 1:** Statistics of *Pseudomonas* sp. A-2 genome assembly and annotation.

Items	Oxford Nanopore Technology	Illumina
Total read base pairs (bp)	1,255,212,065	2,481,461,218
Raw reads	86,002	16,433,518
Trimmed reads	86,002	14,531,470
GC content (%)	59.81	59.84
Contigs	1	21
Contig N50	6,655,306	803,767
Genome length (bp)	6,655,306	6,593,344
CDS	5,896	
rRNA	16	
tRNA	67	
tmRNA	1	

### Pseudomonas sp. A-2 enhances salt stress tolerance in A. thaliana

**Fig. 6:**
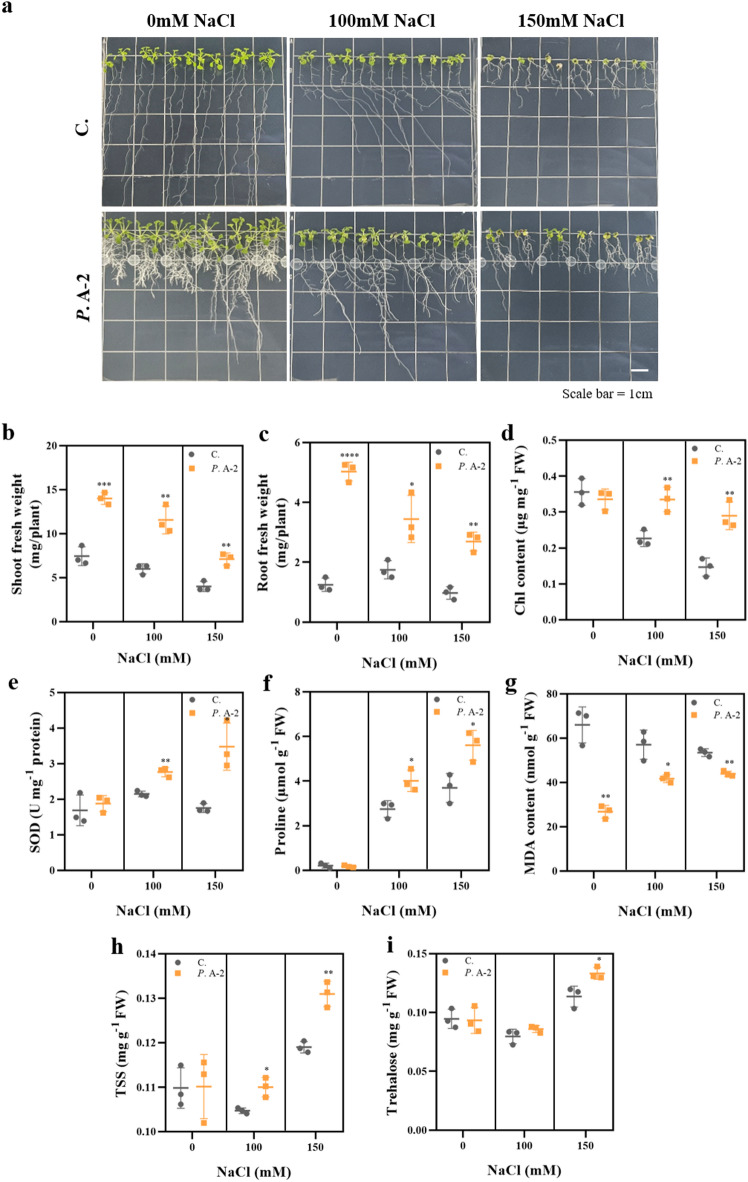
Effects of *Pseudomonas* sp. A-2 on growth performance and physiological responses of *A. thaliana* under salt stress. (**a**) Morphological comparison of *A. thaliana* seedlings grown under different NaCl concentrations (0, 100, and 150 mM) with or without the A-2 strain treatment for 14 days. (b–g) Quantitative measurements of physiological parameters in shoots: shoot fresh weight (**b**), root fresh weight (**c**), total chlorophyll content (**d**), superoxide dismutase (SOD) activity (**e**), proline content (**f**), and malondialdehyde (MDA) content (**g**) of *A. thaliana* seedlings. (**h**–**i**) Soluble sugar content (h, Total Soluble Sugar; mg g^− 1^ FW) and trehalose content (i, mg g^− 1^ FW) under different salt stress conditions. Each bar represents the mean ± SD of three biological replicates. Significant differences between control (C.) and *Pseudomonas* sp. A-2 treated plants (*P*. A-2) were evaluated using Student’s t-test (*P < 0.05, **P < 0.01, ***P < 0.001, ****P < 0.0001).

**Fig. 7:**
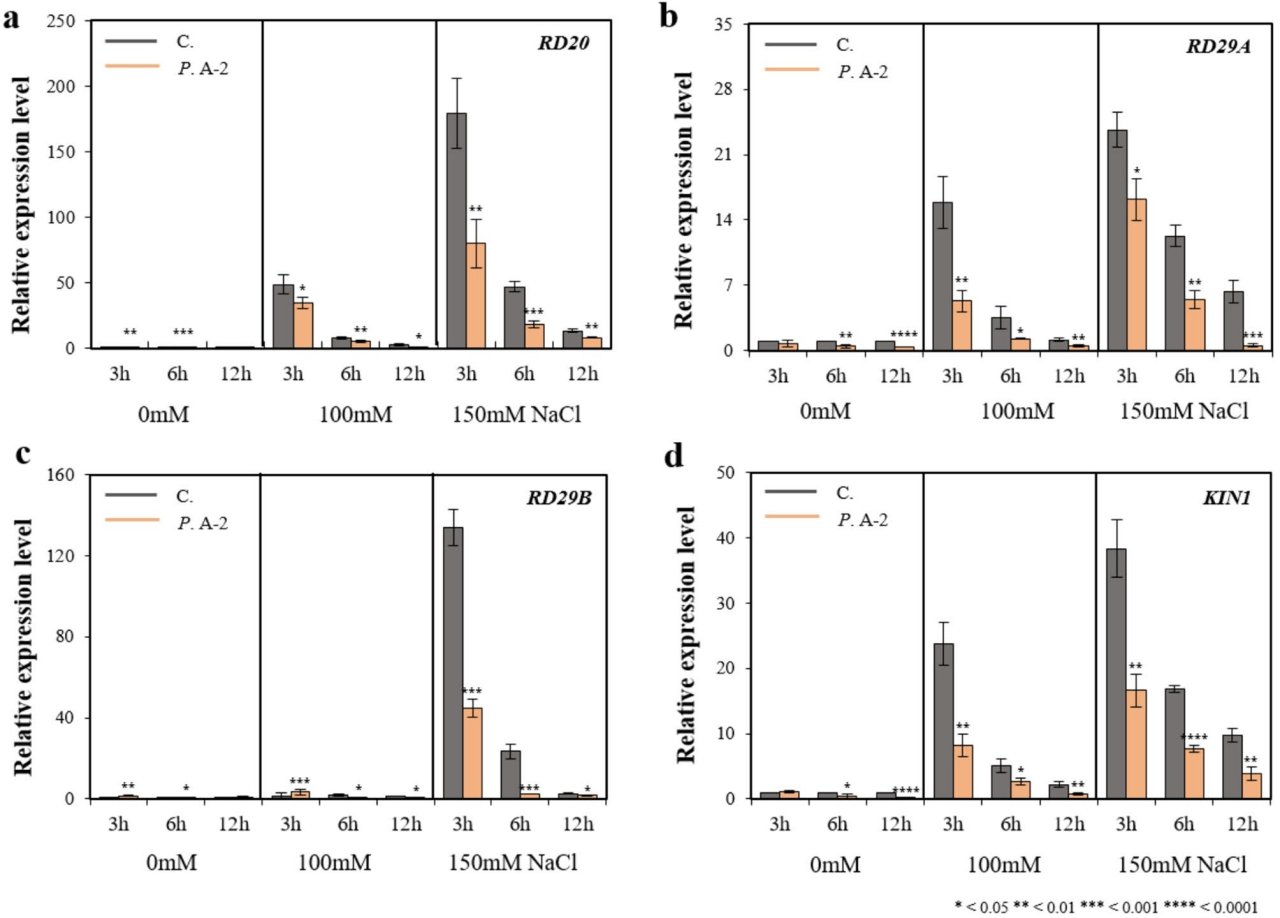
Expression analysis of salt-responsive genes in *A. thaliana* under NaCl stress with or without *Pseudomonas* sp. A-2 treatment. RT-qPCR analysis was conducted on five salt stress–responsive genes: *RD20* (**a**), *RD29A* (**b**), *RD29B* (**c**), and *KIN1* (**d**) in 10-day-old *A. thaliana* seedlings co-cultivated with or without the A-2 strain (OD₆₀₀ = 0.1) under NaCl treatments (0, 100, and 150 mM) for 3, 6, and 12 h. Gene expression levels were calculated using the 2^^–ΔCT^ method and normalized against *actin2* as an endogenous control. Data represent the mean ± standard deviation of three biological replicates. Significant differences between control (C.) and *Pseudomonas* sp. A-2 treated plants (*P*. A-2) were evaluated using Student’s t-test : *P < 0.05, **P < 0.01, ***P < 0.001, ****P < 0.0001. The results are presented as the mean ± standard deviation (*n* = 3).

To evaluate this potential, *A. thaliana* seedlings were cultivated on medium containing 0, 100, or 150 mM NaCl, and physiological as well as molecular responses were assessed after 14 days of the A-2 strain treatment (Figs. 6 and 7). Under non-stress conditions, shoot and root growth were slightly promoted by the A-2 strain treatment (Fig. 6b and c). Under salt stress, growth inhibition was markedly alleviated, as evidenced by significantly higher shoot and root fresh weights in the A-2 strain treated plants compared to untreated controls, indicating improved osmotic stress tolerance. Chlorophyll content, a key indicator of photosynthetic capacity, was better maintained in the A-2 strain treated plants under salt stress (Fig. 6d), suggesting protection of the photosynthetic machinery. In parallel, lipid peroxidation, measured via MDA content, was significantly reduced, indicating mitigation of oxidative stress (Fig. 6g). SOD activity was found to be elevated, particularly at 150 mM NaCl, reflecting enhanced ROS scavenging capacity (Fig. 6e). In support of this observation, antioxidant-related genes such as *katA* (catalase) and *trxC* (thioredoxin) were identified in the A-2 genome (Supplementary Table 1). Catalase is a key enzyme that decomposes hydrogen peroxide (H_2_O_2_) into water and oxygen, thereby protecting cells from oxidative damage^102^. Thioredoxin also plays a crucial role in maintaining redox homeostasis under stress conditions^103^. The presence of these genes suggests that the A-2 strain may directly contribute to enhanced ROS detoxification capacity in the plant through microbial enzymatic activity or redox-modulating signals.

Several salt stress–related genes, including *opuAA*, *opuAB*, *osmY*, *osmV*, and *betB*, were identified in the genome of the A-2 strain (Table 2). These genes are known to be involved in the transport and biosynthesis of osmoprotectants. For example, *opuAA* and *opuAB* are predicted to encode components of the OpuA ABC transporter system, which facilitates the uptake of glycine betaine. This compound functions as an important osmoprotectant in *Bacillus subtilis* under hyperosmotic stress conditions^104^. In addition, the presence of *betB* suggests a possible capacity for the endogenous synthesis of glycine betaine^105^. Taken together, these genomic features suggest that the A-2 strain may contribute to salt stress alleviation by producing compatible solutes and antioxidative metabolites. Furthermore, several regulatory genes were annotated in the A-2 genome, which may be involved in microbial signal transduction or the transcriptional regulation of stress responses. Among them, genes predicted to encode helix-turn-helix (HTH) type transcriptional regulators such as *zntR*, *benM*, *betI*, and *yddM* were identified^106^ (Supplementary Table 1). A gene encoding a sensor histidine kinase, *rcsC*, which functions as part of a two-component regulatory system, was also detected. These regulatory components have been reported to mediate environmental sensing and gene expression control in bacteria^106,107^and may also contribute to the modulation of plant–microbe interactions. Although plant gene expression was not directly assessed in this study, the presence of these elements supports the possibility that the A-2 strain may influence host stress signaling pathways through microbial sensing systems or metabolite-mediated interactions.

**Table 2:** List of genes associated with plant growth–promoting rhizobacteria (PGPR) activity identified in *Pseudomonas* sp. A-2.

PGPR activity types	Gene name	Gene annotation	Start	End	Strand
Indole-3-acetic acid production	*iaaM_1*	Tryptophan 2-monooxygenase	5,261,139	5,263,082	+
	*iaaM_2*	Tryptophan 2-monooxygenase	5,889,969	5,891,663	+
	*trpA*	Tryptophan synthase alpha chain	6,293,550	6,294,362	+
	*trpB*	Tryptophan synthase beta chain	6,292,330	6,293,550	+
	*trpC*	Indole-3-glycerol phosphate synthase	5,828,463	5,829,299	−
	*trpD_1*	Anthranilate phosphoribosyltransferase	4,453,958	4,454,959	+
	*trpD_2*		5,829,296	5,830,351	−
	*trpE*	Anthranilate synthase component 1	5,835,871	5,837,352	−
	*trpF*	*N*-(5’-phosphoribosyl)anthranilate isomerase	4,540,463	4,541,083	−
	*ech1*	Putative enoyl-CoA hydratase 1	5,697,028	5,697,483	+
Phosphate metabolism	*phoU*	Phosphate-specific transport system accessory protein PhoU homolog	6,446,058	6,446,816	+
	*pstB_1*	Phosphate import ATP-binding protein PstB	2,062,402	2,063,181	+
	*pstB_2*		6,445,170	6,446,003	+
	*pstC*	Phosphate transport system permease protein PstC	2,060,532	2,061,527	+
	*pstS_1*	Phosphate-binding protein PstS	535,254	536,288	−
	*pstS_2*		2,059,442	2,060,461	+
	*pstS_3*		6,439,956	6,440,927	+
Nitrogen metabolism	*nasA*	Nitrate transporter	3,704,488	3,705,699	+
Glutamate synthesis	*glnA*	Glutamate synthetase	6,112,204	6,113,610	+
	*gltB*	Glutamate synthase [NADPH] large chain	165,837	170,282	+
	*gltD*	Glutamate synthase [NADPH] small chain	170,417	171,835	+
Siderophore	*rbsR_1*	Lac family transcriptional regulator	648,472	649,485	−
	*rbsR_2*		2,043,431	2,044,429	+
	*rbsR_3*		3,586,499	3,587,551	−
Linear gramicidin synthase	*lgrB*	Linear gramicidin synthase subunit B	4,191,919	4,201,659	−
	*lgrE*	Linear gramicidin dehydrogenase LgrE	6,195,411	6,196,166	+
Salt stress	*opuAA*	Glycine betaine transport ATP-binding protein OpuAA	94,005	94,835	−
	*ousW*	Glycine betaine/choline transport system permease protein OusW	94,832	95,683	−
	*opuAB_1*	Glycine betaine transport system permease protein OpuAB	239,083	239,928	−
	*betA*	Oxygen-dependent choline dehydrogenase	233,617	235,323	−
	*betB*	NAD/NADP-dependent betaine aldehyde dehydrogenase	235,463	236,935	−
	*betI*	HTH-type transcriptional regulator BetI	237,011	237,604	−
	*osmY_1*	Osmoprotectant import permease protein OsmY	782,616	783,329	+
	*osmX_1*	Osmoprotectant-binding protein OsmX	783,356	784,324	+
	*osmW_1*	Osmoprotectant import permease protein OsmW	784,321	784,974	+
	*osmV_1*	Osmoprotectant import ATP-binding protein OsmV	784,971	786,140	+
	*nhaA_1*	Na(+)/H(+) antiporter NhaA	104,018	105,196	−

While only SOD activity was assessed in this study, further analyses of antioxidant-related genes and enzymes would help elucidate the underlying molecular mechanisms. Additionally, proline levels, which contribute to osmotic adjustment, were significantly increased in the A-2 strain treated plants under salt stress (Fig. 6f). Furthermore, total soluble sugars, including trehalose, which contribute to osmotic regulation and help protect cells under salt stress, were significantly increased in the A-2 strain treated plants compared to controls (Fig. 6h and i). These results suggest that the A-2 strain enhances salt stress tolerance in *A. thaliana* by promoting the accumulation of key osmoprotectants that support cellular osmotic balance and stress mitigation.

At the transcriptional level, salt stress was found to strongly induce the expression of *RD20*, *RD29A*, *RD29B*, and *KIN1* in untreated control plants, whereas the induction of these genes was attenuated in the A-2 strain treated plants (Fig. 7). In previous studies, it was demonstrated that plants deficient in *RD29A* and *RD29B* genes show a greater increase in biomass under salt stress^108^. Therefore, the results of this study suggest that the reduced expression of *RD29A* and *RD29B* in the JBR18-treated group may contribute to the enhanced biomass accumulation ability of *Arabidopsis* under salt stress. This indicates that stress signaling was modulated, and transcriptional stress responses were attenuated by the A-2 strain, consistent with previous reports suggesting that PGPR mitigate salt stress by enhancing antioxidant defenses, modulating stress-responsive gene expression, and promoting the accumulation of osmoprotectants^109^.

Collectively, these findings demonstrate that salt stress tolerance in *A. thaliana* was enhanced by the A-2 strain through the protection of cellular functions, reinforcement of antioxidant defenses, and modulation of stress-inducible gene expression. Although osmotic and drought stress conditions were also tested, no consistent or significant improvements were observed in the A-2 strain treated plants under these conditions (data not shown). These results suggest that the stress-alleviating effects of the A-2 strain may be selectively exerted under salt stress conditions, possibly reflecting strain-specific stress response mechanisms.

## Conclusions

In this study, whole-genome sequencing and functional characterization of the novel A-2 strain was conducted, revealing its potential as an eco-friendly biofertilizer. The A-2 strain was identified as a new taxon within the *Pseudomonas* genus and was shown to activate key genetic pathways involved in root development by promoting IAA biosynthesis and inducing the expression of genes associated with both lateral and adventitious root formation. In addition to promoting growth under non-stress conditions, the A-2 strain enhanced *A. thaliana* tolerance to salt stress by improving biomass accumulation, chlorophyll content, antioxidant enzyme activity, proline levels, and the accumulation of total soluble sugars, including trehalose, which function as osmoprotectants. Lipid peroxidation was reduced, and the expression of salt-responsive genes was modulated, indicating improved physiological adaptation and a role in stress signal regulation. Collectively, these findings demonstrate the multifaceted plant growth–promoting and stress-alleviating properties of the A-2 strain, underscoring its potential as a sustainable microbial agent to enhance crop productivity and resilience under adverse environmental conditions.

## Supplementary Information

Below is the link to the electronic supplementary material.

Supplementary Material 1

Supplementary Material 2

## Data Availability

The genome assembly of the strain named Pseudomonas sp. A-2 has been deposited in the Korea Bioinformation Center (https://www.kobic.re.kr/kobic), Korean Read Archive (KRA), under accession ID KAE23766264 and KAE23766265. The strain was deposited as a patent microorganism at the Korean Collection for Type Cultures (KCTC), and the deposit number is KCTC19172P.

## Abbreviations

3DW: Sterile triple-distilled water
AAI: Average Amino Identity
AAO1: Arabidopsis aldehyde oxidase 1
ABP1: Auxin-binding protein 1
AMI1: Amidase 1
ANI: Average nucleotide identity
AR: Adventitious root
ARF: Auxin response factor
ARR1: Arabidopsis response regulator 1
DAT: Days after treatment
dDDH: Digital DNA-DNA hybridization
ECH2: Enoyl-CoA hydratase 2
GGDC: Genome-to-genome distance calculator
IAA: Indole-3-acetic acid
IAM: Indole-3-acetamide
IAOX: Indole-3-acetaldoxime
IBA: Indole-3-butyric acid
IBR: IBA response
IPA: Indole-3-pyruvic acid
L-Trp: L-tryptophan
LR: Lateral root
MDA: Malondialdehyde
MS: Murashige and skoog medium
PDA: Potato dextrose agar
PGPR: Plant growth–promoting rhizobacteria
PIN: Pin-formed proteins
PR: Primary root
SEM: Scanning electron microscopy
SOD: Superoxide dismutase
TAA: Tryptophan aminotransferase of arabidopsis
TAM: Tryptamine
TOB1: Transporter of IBA 1
UPLC: Ultra-performance liquid chromatography
VOC: Volatile organic compound
WGS: Whole-genome sequencing
YUC: YUCCA
