# Correction: Functional characterization of a novel plant growth-promoting rhizobacterium enhancing root growth and salt stress tolerance

**DOI:** 10.1038/s41598-025-24636-x

**Published:** 2025-10-22

**Authors:** Sanghee Lee, Young Kook Kim, Hualin Nie, Jongmin Ahn, Nayoung Kim, Seo-Rin Ko, Ah-Hyeon Choi, Hayoung Kwon, Yuxin Peng, Suk-Yoon Kwon, Ah-Young Shin

**Affiliations:** 1 Plant Systems Engineering Research Center, Korea Research Institute of Bioscience and Biotechnology (KRIBB), Daejeon, 34141 Republic of Korea; 2 Department of Functional Genomics, KRIBB School of Bioscience, University of Science and Technology (UST), 217 Gajeong-ro ,Yuseong-gu, Daejeon, 34113 Republic of Korea; 3 Natural Product Research Center, KRIBB, 30-Yeongudanji-ro, Ochang-eup, Cheongwongu, Cheongju-si, Chungbuk 28116 Republic of Korea; 4 Biological Resource Center, Korean Collection for Type Cultures (KCTC), KRIBB, Jeongeup, 56212 Republic of Korea; 5 Department of Biological Sciences, Sungkyunkwan University, 2066 Seobu-ro, Suwon, 16419 Republic of Korea

Correction to: *Scientific Reports* 10.1038/s41598-025-14065-1, published online 19 August 2025

The original version of this Article contained an error in the name of the author Hualin Nie, which was incorrectly given as Haulin Nie. The original Article has been corrected.

