# Supplementary material for: Functional characterization of a novel plant growth-promoting rhizobacterium enhancing root growth and salt stress tolerance
Source: Sci Rep. 2025 Aug 19;15:30405. doi: 10.1038/s41598-025-14065-1 (PMC12365279; doi:10.1038/s41598-025-14065-1)
Supplement: Supplementary file 2 — Supplementary Material 2 [file 41598_2025_14065_MOESM2_ESM.docx]

**Supplementary Figures**


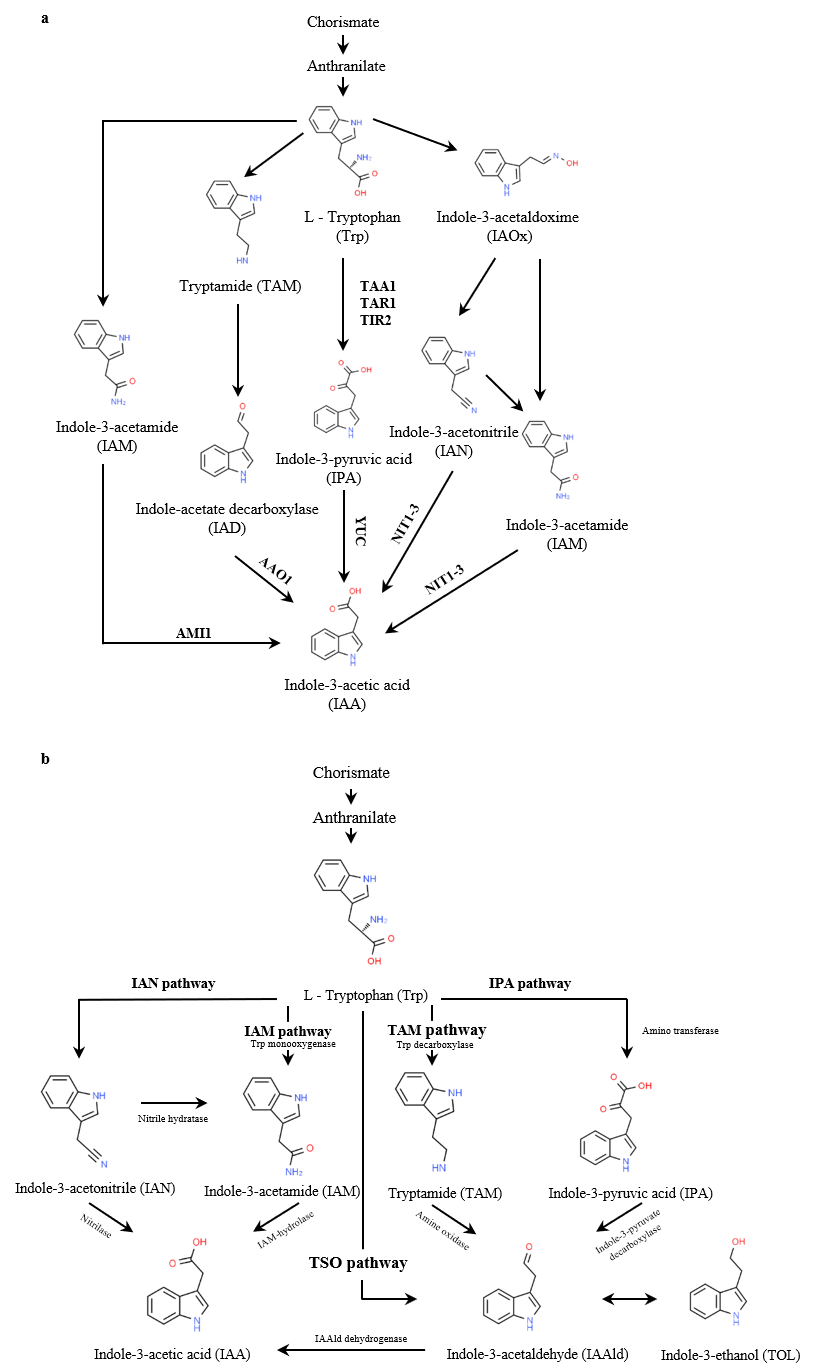


**Supplementary Figure 1.** Simplified diagram of the biosynthetic pathway through which indole-3-acetic acid (IAA) is derived from L-tryptophan in (a) plants and (b) bacteria. Each pathway illustrates the enzymatic conversions leading to the production of IAA, with intermediate compounds shown. AAO1, *Arabidopsis* aldehyde oxidase; AMI1, amidase 1; IAAld, indole-3-acetaldehyde; IAM, indole-3-acetamide; IAN, indole-3-acetonitrile; IAOX, indole-3-acetaldoxime; IPA, indole-3-pyruvic acid; TAA1, tryptophan aminotransferase of *Arabidopsis* 1; TAM, tryptamine; TAR1, tryptophan aminotransferase–related protein 1; TIR2, transport inhibitor response 2; TOL; indole-3-ethanol; TSO, tryptophan side-chain oxidase; YUC, YUCCA.

**
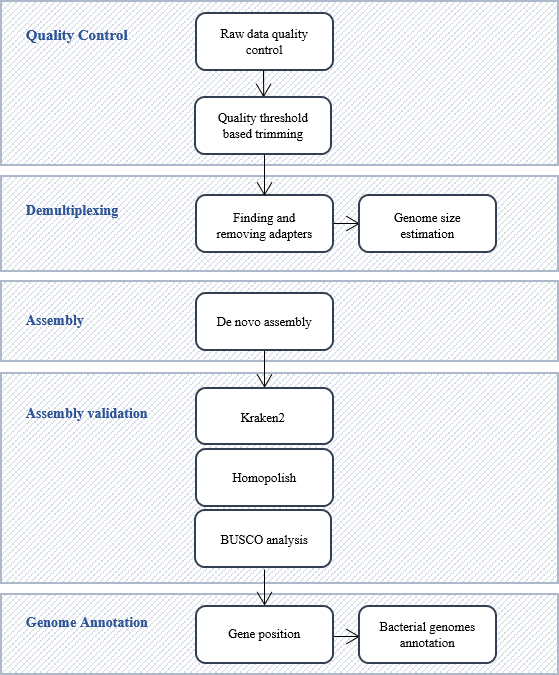
**

**Supplementary Figure 2.** Workflow of ONT sequencing. The process begins with quality control, during which raw data quality is assessed, and low-quality sequences are trimmed based on quality thresholds. During the demultiplexing step, adapters are identified and removed, and genome size estimation is performed. The assembly stage involves the *de novo* assembly of processed reads. Assembly validation includes taxonomic classification with Kraken2, error correction with Homopolish, and completeness assessment with BUSCO analysis. Finally, genome annotation identifies gene positions and annotates bacterial genomes. Each step ensures the integrity and accuracy of the genomic assembly and annotation.


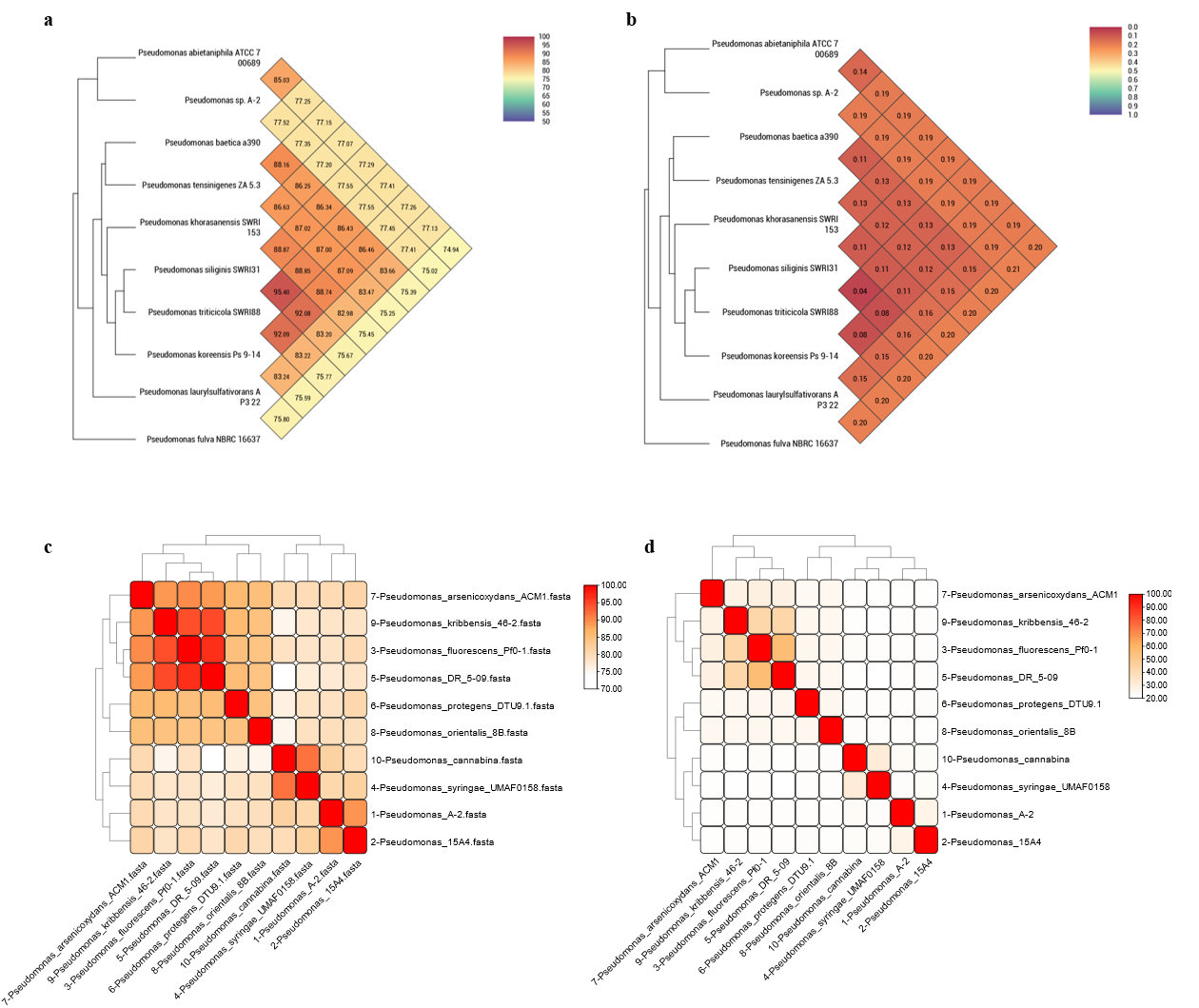


**Supplementary Figure 3.** Comparative genomic analyses of *Pseudomonas* sp. A-2 and related *Pseudomonas* species. (a) Average Nucleotide Identity (ANI) heatmap and hierarchical clustering of the A-2 strain and ten phylogenetically related *Pseudomonas* strains. (b) Genome-to-Genome Distance Calculator (GGDC), shown as a heatmap and hierarchical tree. Both ANI and GGDC analyses were performed using the Orthologous Average Nucleotde Identity tool (OAT, standalone version). (c) Average Amino Identity (AAI) based on gene presence/absence profiles among the analysed *Pseudomonas* genomes, calculated using ezAAI v1.2.3. (d) Pairwise dDDH similarity heatmap among the analysed genomes, generated using GGDC v3.0. Core genome and dDDH analyses further highlight the unique genomic profile of the A-2 strain within the genus.

**
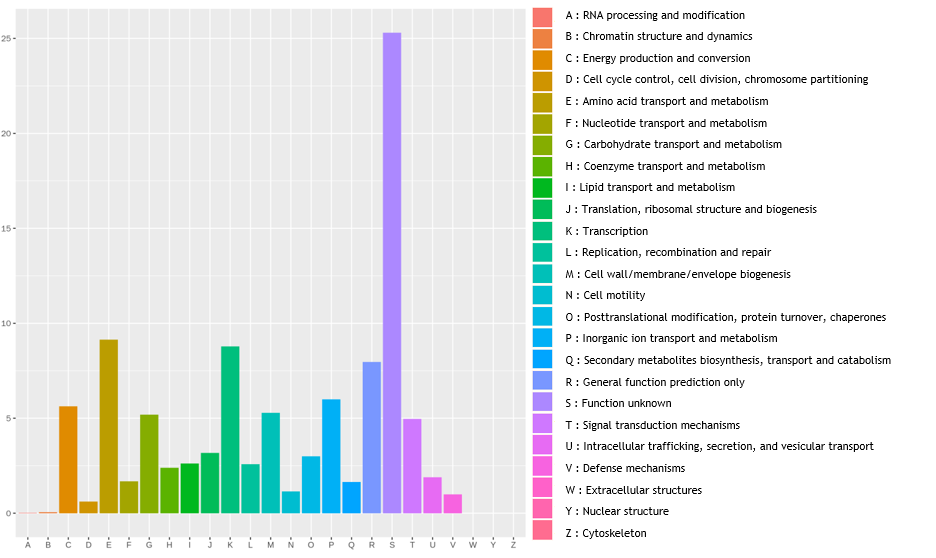
**

**Supplementary Figure 4.** EggNOG category distribution of functional annotation results for *Pseudomonas* sp. A-2. The X-axis represents the 23 different EggNOG functional categories, and the Y-axis shows the proportion of unigenes associated with each category.


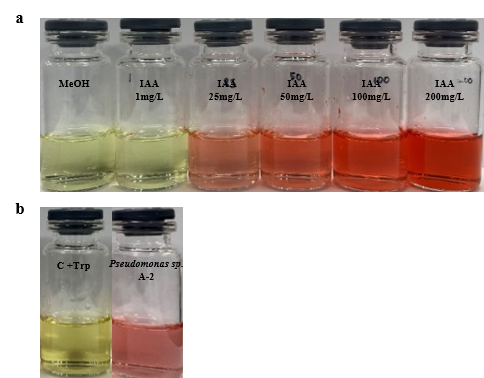


**Supplementary Figure 5.** Photographic representation of the synthetic IAA concentration standard and the production of IAA by *Pseudomonas* sp. A-2 in the presence of tryptophan (100 mg/mL), as visualized with Salkowski’s reagent. (a) Synthetic IAA solutions were prepared at concentrations of 1, 25, 50, 100, and 200 mg/L. Upon the addition of Salkowski’s reagent, the presence of IAA is indicated by the development of a pink color, which intensifies with increasing IAA concentrations. (b) Comparison of a control containing tryptophan only (C+Trp) and a sample from the A-2 strain cultured in the presence of 100 mg/mL tryptophan. The observed pink color in the A-2 sample indicates IAA production by the bacterial strain.


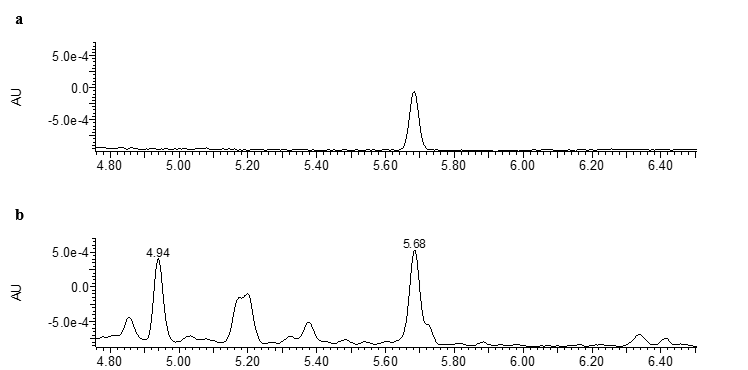


**Supplementary Figure 6.** Ultra-performance liquid chromatography–ultraviolet analysis of IAA extraction. (a) Chromatogram of the IAA standard (1.56 ppm) at a wavelength of 280 nm, showing a distinct peak with a retention time of approximately 5.68 minutes. (b) Chromatogram of IAA extracted from *Pseudomonas* sp. A-2 culture grown in the absence of tryptophan, with retention times detected at a wavelength of 280 nm. Peaks in (b) include one matching the IAA standard retention time at 5.68 minutes, indicating IAA production by the A-2 strain.
